# Increased Proteinuria is Associated with Increased Aortic Arch Calcification, Cardio-Thoracic Ratio, Rapid Renal Progression and Increased Overall and Cardiovascular Mortality in Chronic Kidney Disease

**DOI:** 10.7150/ijms.45470

**Published:** 2020-04-27

**Authors:** Wei-Yu Su, Pei-Yu Wu, Jiun-Chi Huang, Szu-Chia Chen, Jer-Ming Chang

**Affiliations:** 1 Department of General Medicine, Kaohsiung Medical University Hospital, Kaohsiung, Taiwan;; 2 Division of Nephrology, Department of Internal Medicine, Kaohsiung Medical University Hospital, Kaohsiung Medical University, Kaohsiung, Taiwan;; 3 Department of Internal Medicine, Kaohsiung Municipal Siaogang Hospital, Kaohsiung Medical University, Kaohsiung, Taiwan;; 4 Faculty of Medicine, College of Medicine, Kaohsiung Medical University, Kaohsiung, Taiwan; 5 Research Center for Environmental Medicine, Kaohsiung Medical University, Kaohsiung, Taiwan

**Keywords:** proteinuria, aortic arch calcification, cardio-thoracic ratio, rapid renal progression, overall mortality, cardiovascular mortality, chronic kidney disease

## Abstract

**Background**: Patients with chronic kidney disease (CKD) are associated with high prevalence rates of proteinuria, vascular calcification and cardiomegaly. In this study, we investigated relationships among proteinuria, aortic arch calcification (AoAC) and cardio-thoracic ratio (CTR) in patients with CKD stage 3A-5. In addition, we investigated correlations among proteinuria and decline in renal function, overall and cardiovascular (CV) mortality.

**Methods**: We enrolled 482 pre-dialysis patients with CKD stage 3A-5, and determined AoAC and CTR using chest radiography at enrollment. The patients were stratified into four groups according to quartiles of urine protein-to-creatinine ratio (U_PCR_).

**Results**: The patients in quartile 4 had a lower estimated glomerular filtration rate (eGFR) slope, and higher prevalence rates of rapid renal progression, progression to commencement of dialysis, overall and CV mortality. Multivariable analysis showed that a high U_PCR_ was associated with high AoAC (unstandardized coefficient β: 0.315; *p* = 0.002), high CTR (unstandardized coefficient β: 1.186; *p* = 0.028) and larger negative eGFR slope (unstandardized coefficient β: -2.398; *p* < 0.001). With regards to clinical outcomes, a high U_PCR_ was significantly correlated with progression to dialysis (log per 1 mg/g; hazard ratio [HR], 2.538; *p* = 0.003), increased overall mortality (log per 1 mg/g; HR, 2.292; *p* = 0.003) and increased CV mortality (log per 1 mg/g; HR, 3.195; *p* = 0.006).

**Conclusions**: Assessing proteinuria may allow for the early identification of high-risk patients and initiate interventions to prevent vascular calcification, cardiomegaly, and poor clinical outcomes.

## Introduction

Patients with chronic kidney disease (CKD) are at a high risk of end-stage renal disease (ESRD) and cardiovascular (CV) and all-cause mortality 1. Patients with CKD are associated with many subclinical CV risk factors that may cause rapid renal progression, CV and overall mortality. One of the most important markers for progressive renal function decline, CV and overall mortality in CKD patients is proteinuria 2. Many studies have reported an association between elevated albuminuria and CV disease, even if albuminuria is at the upper end of the normal range (threshold 30 mg/g) 3. In addition, vascular calcification (VC) in the aorta has been reported to be a risk factor for CV morbidity and mortality in patients on long-term dialysis 1. VC is commonly seen in patients with CKD or ESRD 4.

Chest radiography is a non-invasive, fast and inexpensive tool used to evaluate aortic arch calcification (AoAC) and cardiothoracic ratio (CTR) in daily practice. AoAC identified by chest radiography has been associated with pulse pressure, intima-media thickness of the common carotid artery, CV events, and an increased risk of CV mortality in the general population 5,6. CTR is an easily obtainable parameter which has been associated with left ventricular size, and a high CTR is considered to represent an increased left ventricular mass (LVM), and even left ventricular hypertrophy 7. In patients undergoing maintenance hemodialysis, a high CTR has been independently associated with a high risk of all-cause mortality and CV events 8. In addition, associations among AoAC and CTR and a decline in renal function have been reported in patients with CKD stage 3-5 9. In our recent study, we also found that increased AoAC and cardiomegaly were associated with a rapid decline in renal function and increased CV mortality in patients with CKD 10.

Nevertheless, associations among the degree of proteinuria and AoAC and CTR in patients with CKD have not been thoroughly investigated. Therefore, the aim of this study was to evaluate relationships among proteinuria and AoAC and CTR in patients with CKD stage 3A-5. In addition, we investigated correlations among proteinuria and a decline in renal function, overall and CV mortality.

## Study Patients and Methods

### Study Patients and Design

We consecutively enrolled 638 pre-dialysis patients diagnosed with CKD stage 3A-5 according to the guidelines of the Kidney Disease Improving Global Outcomes 2012 (KDIGO 2012) 11 from the internal medicine outpatient department of a regional hospital in southern Taiwan from March 2007 to January 2016. The patients all had evidence of kidney damage lasting for > 3 months, and they were classified as having CKD stages 3A, 3B, 4 and 5 according to an estimated glomerular filtration rate (eGFR) of 45 to 59 mL/min/1.73 m^2^, 30 to 44 mL/min/1.73 m^2^, 15 to 29 mL/min/1.73 m^2^, and < 15 mL/min/1.73 m^2^, respectively. We excluded 67 patients who had < three recorded measurements of eGFR during the follow-up period. We also excluded 89 patients who were followed up for < 6 months to avoid incomplete observations of changes in renal function. The remaining 482 patients (mean age 65.5 ± 12.2 years, 283 males) were included in this study. The study protocol was approved by the Institutional Review Board of Kaohsiung Medical University Hospital, and all participants provided written informed consent to participate in this study. The methods were carried out in accordance with the approved guidelines.

### Evaluation of AoAC and CTR by Chest X-Ray

All of the included patients received chest X-rays, which were reviewed by a single experienced radiologist who was blinded to the clinical data of the patients. AoAC was assessed using a scale developed by Ogawa et al. 12 which classifies the aortic arch into 16 sections according to its circumference, and the number of calcified sections was counted and recorded for each patient. CTR was also calculated from the X-rays as the transverse diameter of the cardiac silhouette divided by that of the chest.

### Demographic, Medical and Laboratory Data

The following baseline demographic, medical and laboratory variables were recorded: age, sex, smoking history (ever *vs.* never), presence of cerebrovascular disease, coronary artery disease, hypertension, diabetes mellitus, body mass index, systolic blood pressure, diastolic blood pressure, levels of triglycerides, total cholesterol, fasting glucose, hemoglobin, total calcium, phosphorous, calcium-phosphorous product, eGFR, uric acid, parathyroid hormone (PTH) and U_PCR_. The use of medications including angiotensin II receptor blockers (ARBs), angiotensin converting enzyme inhibitors (ACEIs) and calcium-based phosphate binders was also recorded. The demographic variables were obtained from baseline records, and the medical data was obtained from a chart review. Fasting blood and urine samples were collected from the patients within 1 month of enrollment, and the laboratory data were obtained (COBAS Integra 400, Roche Diagnostics GmbH, D-68298 Mannheim), and the compensated Jaffé method (kinetic alkaline picrate) was used to calculate levels of serum creatinine (Roche/Integra 400 Analyzer, Roche Diagnostics) as previously described 13. EGFR was calculated using the Modification of Diet in Renal Disease-4 equation 14.

### Assessment of Decline in Renal Function and Definition of Rapid Renal Progression

The rate of decline in renal function was evaluated using the eGFR slope, which was plotted using at least three measurements and defined as the regression coefficient between eGFR and time. A decline > 3 ml/min/1.73 m^2^/year was defined as rapid renal progression 15. Renal function data were censored in the patients who progressed to renal replacement therapy. The other patients were followed until September 2018.

### Definition of Renal End Point

The renal endpoint was defined as starting dialysis. Renal function data were censored at the initiation of renal replacement therapy for those who reached the endpoint. The other patients were followed until September 2018. The date of starting dialysis was determined according to the regulations for dialysis therapy of the National Health Insurance program in Taiwan, which are based on uremic symptoms and signs, nutrition status, and laboratory data.

### Definition of Overall and CV Mortality

Cases of overall and CV mortality were defined by two cardiologists from medical records. Disagreements were resolved after consultation with a third cardiologist. The patients were followed until death or September 2018, whichever occurred first.

### Reproducibility

The reproducibility of AoAC was evaluated by an experienced radiologist and a medical doctor in 30 patients who were selected at random. The mean percent error was calculated as the difference divided by the average of the two observations, and was 12.3 ± 12.3% in this study.

### Statistical Analysis

Statistical analysis was performed using SPSS 19.0 for Windows (SPSS Inc. Chicago, USA). Data were expressed as percentage, mean ± standard deviation, or median (25^th^-75^th^ percentile) for triglycerides, PTH, U_PCR_ and eGFR slope. The study patients were classified into four groups according to quartiles of U_PCR_. Among-group comparisons were performed using one-way analysis of variance followed by a Bonferroni-adjusted post hoc test. Multivariate stepwise linear regression analysis was used to identify factors associated with AoAC, CTR and eGFR slope. Survival curves for dialysis-free, overall and CV survival were plotted using the Kaplan-Meier method. The time to commencing dialysis, overall and CV mortality and covariates of risk factors were modeled using a multivariable forward Cox proportional hazards model. The patients in quartile 1, who had the lowest risk of mortality, served as the reference group. *P* < 0.05 was considered to indicate a significant difference.

## Results

A total of 482 patients (283 men and 199 women) with CKD stage 3A-5 were included, with a mean age of 65.5 ± 12.2 years. The patients were classified into four groups according to quartiles of U_PCR_. The clinical characteristics of these four groups are shown in Table 1. There were 116, 124, 119 and 123 patients in the four groups, respectively. Compared to the patients in quartile 1, more of those in quartile 4 were female, and they had higher prevalence rates of diabetes mellitus and hypertension, higher systolic blood pressure, higher CTR, higher levels of triglycerides and total cholesterol, lower total calcium, higher phosphorous, higher calcium-phosphorous product, higher PTH, higher U_PCR_, lower hemoglobin, lower baseline eGFR, higher prevalence of advanced CKD stage and higher percentage of calcium-based phosphate binders use. With regards to the outcomes, the patients in quartile 4 had a lower eGFR slope, more rapid renal progression, progression to commencement of dialysis, overall and CV mortality compared to those in quartile 1.

**Figure 1:**
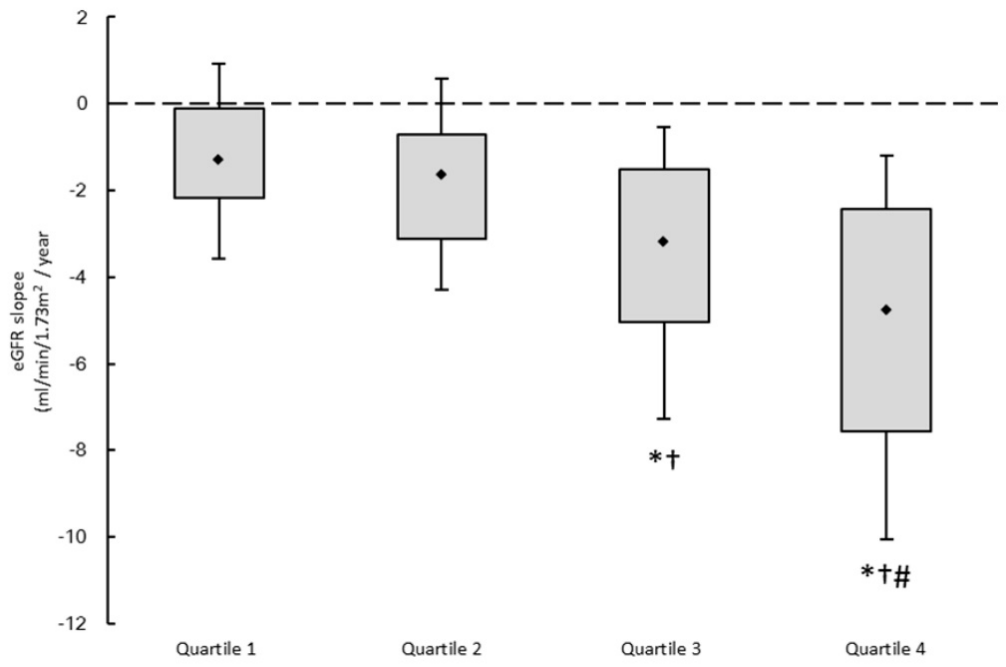
The estimated glomerular filtration rate (eGFR) slopes among 4 study groups. ^*^p < 0.05 compared quartile 1 of U_PCR_; ^†^*p* < 0.05 compared with quartile 2 of U_PCR_; ^#^*p* < 0.05 compared with quartile 3 of U_PCR_.

Figure 1 illustrates the eGFR slopes of the four study groups, with median values of -1.31, -1.64, -3.18, and -4.78 mL/min/1.73 m^2^/year, respectively. The patients in quartile 4 had the lowest eGFR slope.

### Determinants of AoAC

Table 2 shows the determinants of AoAC in all patients. In the multivariate stepwise linear regression analysis after adjusting for age, sex, body mass index, smoking status, cerebrovascular disease, coronary artery disease, hypertension, systolic and diastolic blood pressures, diabetes mellitus, fasting glucose, hemoglobin, log-transformed triglycerides, total cholesterol, hemoglobin, baseline eGFR, total calcium, phosphorous, calcium-phosphorous product, uric acid, PTH, log-transformed U_PCR_, ACEI/ARB antihypertensive drug and calcium-based phosphate binders use, old age, high calcium-phosphorous product, low PTH, and high U_PCR_ (unstandardized coefficient β: 0.315; 95% confidence interval [CI], 0.119 to 0.511; *p* = 0.002) were independently correlated with high AoAC.

### Determinants of CTR

Table 3 shows the determinants of CTR in all patients. In the multivariate stepwise linear regression analysis, old age, male sex (*vs.* female), coronary artery disease, cerebrovascular disease, high body mass index, low hemoglobin, high uric acid, high U_PCR_ (unstandardized coefficient β: 1.186; 95% CI, 0.130 to 2.242; *p* = 0.028), and calcium-based phosphate binders use were independently associated with high CTR.

### Determinants of eGFR slope

Table 4 shows the determinants of eGFR slope in all patients. In the multivariate stepwise linear regression analysis, low diastolic blood pressure, high uric acid, and high U_PCR_ (unstandardized coefficient β: -2.398; 95% CI, -2.965 to -1.831; *p* < 0.001) were independently correlated with larger negative values of the eGFR slope.

### Risk of Progression to Dialysis

The follow-up period was 4.4 (2.8-7.3) years, during which 167 patients (34.6%) started hemodialysis. Table 5 shows the hazard ratios (HRs) for age, sex, body mass index, smoking status, cerebrovascular disease, coronary artery disease, hypertension, systolic and diastolic blood pressures, diabetes mellitus, fasting glucose, log-transformed triglyceride, total cholesterol, hemoglobin, baseline CKD stage, total calcium, phosphorous, calcium-phosphorous product, uric acid, PTH, log-transformed U_PCR_, ACEI/ARB antihypertensive drug and calcium-based phosphate binders use, and quartiles of U_PCR_ (model 1) or log-transformed U_PCR_ (model 2). The multivariate regression analysis (model 1) showed that compared to those in quartile 1 of U_PCR_, those in quartile 3 (HR, 6.731; 95% CI, 1.531 to 29.600; *p* = 0.012), and quartile 4 (HR, 6.639; 95% CI, 1.466 to 30.075; *p* = 0.014) were significantly associated with progression to dialysis. In the multivariate regression analysis (model 2), increased U_PCR_ (log per 1 mg/g; HR, 2.538; 95% CI, 1.375 to 4.685; *p* = 0.003) was significantly correlated with progression to dialysis.

**Figure 2:**
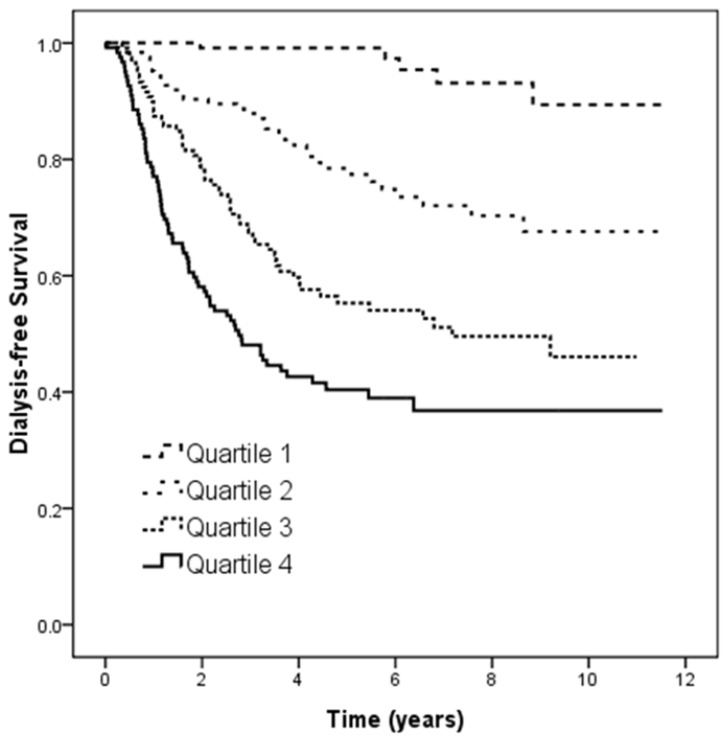
Kaplan-Meier analyses of dialysis-free survival (log-rank *p* < 0.001) among 4 study groups. The group with quartile 2, quartile 3, and quartile 4 of U_PCR_ had worse dialysis-free survival than that with quartile 1 of U_PCR_.

Figure 2 illustrates the Kaplan-Meier analysis of dialysis-free survival (log-rank *p* < 0.001) among four study groups. The patients in quartiles 2, 3, and 4 of U_PCR_ had worse dialysis-free survival than those in quartile 1.

### Risk of Overall Mortality

The median follow-up period was 5.7 (3.6-8.0) years, during which 86 of the 482 patients died (17.8%) due to CV causes (n = 31), malignancy (n = 7), infectious diseases (n = 40), gastrointestinal bleeding (n = 4), and others (n = 4). The multivariate regression analysis (model 1) showed that compared to the patients in quartile 1 of U_PCR_, those in quartile 3 (HR, 3.452; 95% CI, 1.179 to 10.109; *p* = 0.024), and quartile 4 (HR, 4.845; 95% CI, 1.805 to 12.999; *p* = 0.002) were significantly associated with increased overall mortality (Table 5). Further, model 2 of the multivariate regression analysis showed that increased U_PCR_ (log per 1 mg/g; HR, 2.292; 95% CI, 1.329 to 3.953; *p* = 0.003) was also significantly associated with increased overall mortality.

**Figure 3:**
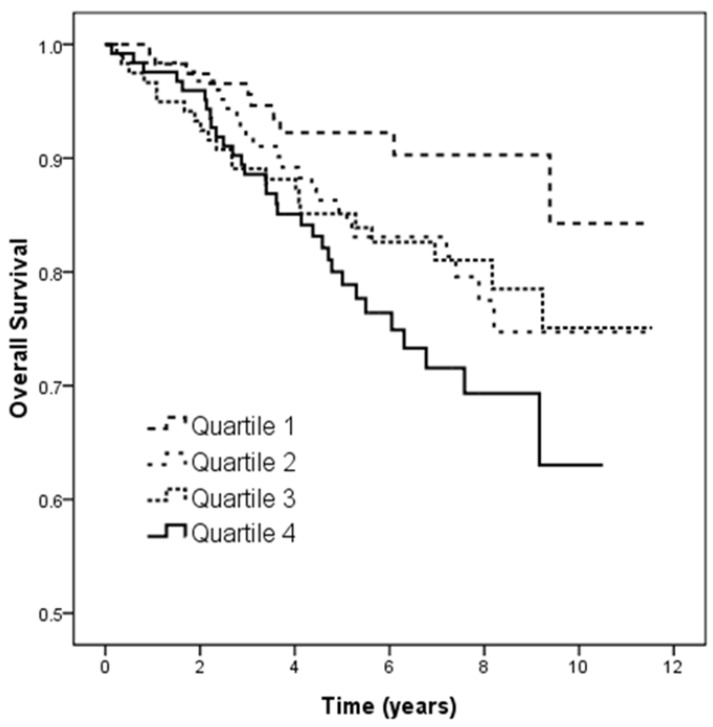
Kaplan-Meier analyses of overall survival (log-rank *p* = 0.021) among 4 study groups. The group with quartile 4 of U_PCR_ had worse overall survival than that with quartile 1 of U_PCR_.

Figure 3 illustrates the Kaplan-Meier analysis of overall survival (log-rank *p* = 0.021) among the four study groups. The patients in quartile 4 of U_PCR_ had worse overall survival than those in quartile 1.

### Risk of CV Mortality

The 31 patients who died due to CV causes during follow-up included heart failure (n = 8), myocardial infarction (n = 6) and ventricular fibrillation (n = 17). Multivariate forward Cox proportional hazards regression analysis of CV mortality in the four study groups is shown in Table 5. The patients in quartile 3 of U_PCR_ (HR, 11.741; 95% CI, 1.422 to 96.935; *p* = 0.022) and quartile 4 (HR, 12.974; 95% CI, 1.642 to 102.481; *p* = 0.015) (*vs.* quartile 1 of U_PCR_) in model 1, and increased U_PCR_ (log per 1 mg/g; HR, 3.195; 95% CI, 1.393 to 7.325; *p* = 0.006) in model 2 were significantly associated with increased CV mortality.

**Figure 4:**
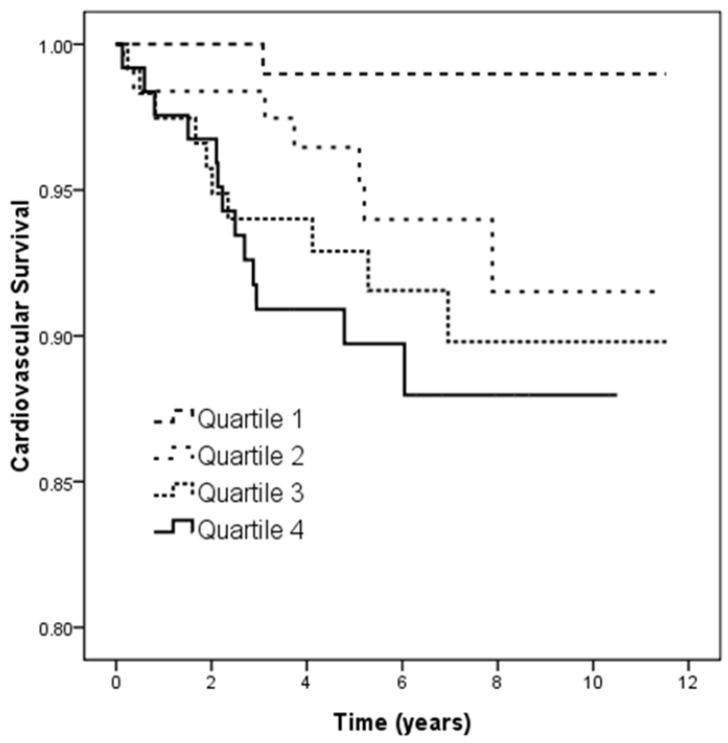
Kaplan-Meier analyses of cardiovascular survival (log-rank *p* = 0.019) among 4 study groups. The group with quartile 3 and quartile 4 of U_PCR_ had worse overall survival than that with quartile 1 of U_PCR_.

Figure 4 illustrates the Kaplan-Meier analysis of CV survival (log-rank *p* = 0.019) among the four study groups. The patients in quartile 3 and quartile 4 of U_PCR_ had worse CV survival than those in quartile 1.

## Discussion

This study demonstrated associations among proteinuria and renal function progression, overall and CV mortality in patients with CKD stages 3-5. We found that high U_PCR_ was associated with a rapid decline in renal function, high AoAC and high CTR. In addition, the patients with high U_PCR_ had a higher risk of progression to dialysis, overall and CV mortality.

To the best of our knowledge, this is the first study to report an association between high U_PCR_ and high AoAC as measured from chest X-rays in patients with CKD stages 3-5. Dayan et al. reported an association between coronary artery calcification and albuminuria in patients with type 2 diabetes 16. Similarly, Freedman et al. also reported an association between albuminuria and calcified plaques in coronary and carotid arteries in 588 patients with type 2 diabetes 17. However, a study conducted by Li et al. showed that age, eGFR slope, and increased CTR were independent determinants of AoAC, but that proteinuria was not 9. Another study also reported an independent association between descending thoracic aortic calcium and eGFR, but not with urinary albumin to creatinine ratio (U_ACR_) 18. Thus, the status of proteinuria as a risk marker for VC remains controversial. In CKD, interstitial capillaries become increasingly permeable, allowing plasma proteins to reach the renal interstitium and trigger an inflammatory response. In vitro, exposure of proximal tubular cells to plasma proteins including albumin, transferrin and IgG has been shown to result in the release of pro-inflammatory and pro-fibrotic molecules including interleukin-8 (IL-8), NF-kappaB, endothelin-1(ET-1), monocyte chemoattractant protein-1 (MCP-1), regulated on activation normal T expressed and secreted (RANTES) chemokine, fractalkine, and osteopontin 19-22. Moreover, ET-1, NF-kappaB, interleukin (IL)-8 and osteopontin have been shown to be potent regulators of VC in vivo 23-26. VC can occur in the tunica intima or tunica media of the arterial wall, or both, and the clinical consequences of intimal versus medial layer calcification can be quite different. For example, intimal calcification is associated with plaque rupture and acute vessel occlusion, whereas increased arterial stiffness is associated with medial calcification 27. Inflammation and oxidative stress are involved in the process of VC 10. Inflammatory cytokines such as tumor necrosis factor and IL-6 have been reported to induce the differentiation of vascular smooth muscle cells and VC. In addition, inflammation has also been associated with the production of reactive oxygen species, which can then further induce vascular remodeling and VC 10. Moreover, proteinuria promotes the inflammation mediated by these cytokines, leading to increased AoAC.

Another important finding of this study is that a high U_PCR_ were associated with a high CTR in the patients with CKD stages 3-5. Previous studies have assessed LVM using electrocardiography, echocardiography, and magnetic resonance imaging 28-30. The MONICA/KORA study demonstrated that even low levels of albuminuria was a significant predictor of LVM assessed using echocardiography in the general population 28. Using Cornell electrocardiographic voltage criteria, Nobakhthagighi et al. reported associations among LVM, urinary albumin excretion and mortality in patients with type 2 diabetes 29. In addition, proteinuria has been independently and significantly associated with LVM as measured by cardiac magnetic resonance imaging in patients with CKD 30. Recently, Matsushita et al. investigated cross-sectional associations between eGFR and albuminuria with LVM in the Atherosclerosis Risk in Communities Study, and found that both higher albuminuria and lower eGFR were independently associated with left ventricular structure and function electrocardiographic parameters 31. The pathophysiology of cardiomegaly in patients with CKD is multifactorial in origin. Patients with CKD often have VC, which can lead to increased systemic arterial resistance, higher arterial blood pressure, and a reduction in large-vessel compliance, resulting in myocardial cell thickening and concentric remodeling of the left ventricle 32. As mentioned, proteinuria, left ventricular hypertrophy and VC interact with each other and share common pathways including oxidative stress. In addition, Chen et al. demonstrated that CTR could be an indicator of inflammation in patients without diabetes on hemodialysis 33.

The third important finding in this study is that a high U_PCR_ was associated with a rapid decline in renal function and a higher risk of progression to dialysis in the patients with CKD stages 3-5. Tubular atrophy, interstitial fibrosis and scarring are closely associated with glomerular filtration rate and proteinuria. The abnormal filtration of various urinary proteins including cytokines, complement, and albumin can stimulate tubular epithelial cells to produce inflammatory products including chemokines and reactive oxygen species. This then causes inflammatory cells to enter the renal interstitium and interact with interstitial myofibroblasts. As fibrosis develops, injured tubular epithelia are no longer able to regenerate and undergo apoptosis, thereby leading to tubular atrophy with nonfunctioning glomeruli 34.

Previous studies have reported a strong association between proteinuria and the risk of CKD progression 35-38. A study involving 107,192 participants in Okinawa, Japan, identified proteinuria as the most powerful predictor of the risk of ESRD over 10 years in the general population 35. The African-American Study of Kidney Disease and Hypertension (AASK) included patients without diabetes but with CKD and found that a higher baseline proteinuria level was associated with a faster decline in glomerular filtration rate 36. In addition, among patients with diabetic nephropathy, baseline U_ACR_ was a strong independent predictor of ESRD in the Reduction of Endpoints in NIDDM with the Angiotensin II Antagonist Losartan (RENAAL) study and in the Irbesartan in Diabetic Nephropathy Trial (IDNT) 37, 38. Moreover, Inker et al. reported that an early reduction in proteinuria was associated with a slower progression of kidney disease, and that this association was stronger in the patients with higher levels of baseline proteinuria 39.

Another finding of this study is that a high U_PCR_ was associated with a higher risk of overall and CV mortality in the patients with CKD stages 3-5. This is consistent with previous studies which reported an association between proteinuria and overall and CV mortality 40, 41. A large meta-analysis of general population cohorts analyzed 21 studies with more than 100,000 individuals with U_ACR_, and found that the relationship between albuminuria and mortality was linear on a log-log scale, with a 2-fold higher risk at a U_ACR_ of approximately 100 mg/g compared to an optimal U_ACR_ level (5 mg/g) independently of eGFR and conventional risk factors 40. Another systematic review and meta- and pooled analyses of seven prospective cohorts in Japan demonstrated that proteinuria was associated with a 1.75-fold (95% CI): 1.44, 2.11) increased risk of CV disease mortality after adjusting for potential confounding factors 41. In addition, patients with both proteinuria and a eGFR of <45 mL/minute/1.73 m^2^ had a 4.05-fold (95% CI: 2.55, 6.43) higher risk of CV disease mortality compared to those with neither of these risk factors. The association between microalbuminuria and CV disease may be due to common pathophysiologic processes, such as endothelial dysfunction or chronic low-grade inflammation 42. The present study clearly showed that proteinuria was associated with the risk of overall and CV disease mortality, which supports the view that assessing proteinuria is needed to improve the identification of individuals at high risk of CV complications and to establish appropriate preventative measures for these patients.

There are several limitations to this study. First, the study patients were enrolled from one regional hospital in southern Taiwan, and thus, the generalizability of our results is limited. Second, this was an observational study, and there were variations in the frequency and number of laboratory examinations between patients, including serum creatinine measurements. To minimize this effect, we excluded patients who were followed for less than 6 months or had fewer than three eGFR measurements during the follow-up period. Third, only one radiologist assessed CTR and AoAC on the chest radiographs, and therefore observation bias may have existed. We tested the reproducibility of AoAC by having another trained medical doctor screen some of the chest plain films and calculating the mean percent error. Fourth, CTR and AoAC were measured only once at enrollment. Therefore, the association between the effect of AoAC and CTR over time could not be estimated.

In conclusion, this is the first study to report an association between a high U_PCR_ and high AoAC and CTR in patients with CKD stages 3-5. Furthermore, a high degree of proteinuria was correlated with a rapid decline in renal function, the risk of progression to dialysis, and overall and CV mortality. Assessments of proteinuria may be beneficial to allow for the early identification of high-risk patients and initiate interventions to prevent VC, cardiomegaly, rapid decline in renal function, progression to dialysis, and to increase overall, dialysis-free and CV survival.

## Figures and Tables

**Table 1:** Comparison of clinical characteristics according to quartiles of urine protein-to-creatinine ratio (Upcr)

Characteristics	Quartile 1 (n = 116)	Quartile 2 (n = 124)	Quartile 3 (n = 119)	Quartile 4 (n = 123)	*p*
Age (year)	67.7 ± 12.8	64.7 ± 12.4	65.8 ± 12.5	63.8 ± 11.1	0.074
Male gender (%)	67.2	65.3	57.1	45.5^*†^	0.002
Smoking (%)	29.6	33.1	28.6	20.3	0.149
Diabetes mellitus (%)	45.7	48.4	57.6	82.9^*†#^	< 0.001
Hypertension (%)	80.2	82.3	91.5	94.3^*†^	0.001
Coronary artery disease (%)	13.8	12.2	14.5	13.0	0.958
Cerebrovascular disease (%)	9.5	12.9	7.6	6.6	0.332
Systolic blood pressure (mmHg)	137.8 ± 19.2	139.4 ± 21.9	146.0 ± 22.4^*^	154.3 ± 24.6^*†#^	< 0.001
Diastolic blood pressure (mmHg)	76.9 ± 13.3	78.1 ± 13.0	79.0 ± 15.3	76.9 ± 14.8	0.620
Body mass index (kg/m^2^)	25.6 ± 3.5	25.3 ± 3.9	25.5 ± 4.0	25.6 ± 4.1	0.926
AoAC	3.5 ± 1.1	3.6 ± 0.8	3.8 ± 0.8	3.8 ± 0.9	0.020
CTR (%)	47.8 ± 5.1	48.3 ± 5.2	50.5 ± 5.8^*†^	51.5 ± 5.8^*†^	< 0.001
Laboratory parameters					
Fasting glucose (mg/dL)	120.3 ± 45.5	124.3 ± 52.6	123.4 ± 53.3	136.9 ± 63.2	0.086
Triglyceride (mg/dL)	121 (90-193.5)	142 (90-205.5)	132 (97-187)	148 (115-208)^*^	0.041
Total cholesterol (mg/dL)	188.4 ± 44.0	197.6 ± 56.8	193.2 ± 52.2	219.0 ± 65.1^*†#^	< 0.001
Hemoglobin (g/dL)	12.6 ± 2.0	11.4 ± 2.3^*^	10.9 ± 2.2^*^	10.0 ± 1.8^*†#^	< 0.001
Baseline eGFR (ml/min/1.73m^2^)	34.5 ± 12.0	25.9 ± 14.4^*^	20.6 ± 12.3^*†^	18.7 ± 10.6^*†^	< 0.001
CKD stage		^*^	^*†^	^*†^	< 0.001
3A (%)	16.4	8.1	2.5	2.4	
3B (%)	44.0	27.4	16.8	11.4	
4 (%)	37.9	37.9	42.0	41.5	
5 (%)	1.7	26.6	38.7	44.7	
Total calcium (mg/dL)	9.4 ± 0.5	9.4 ± 0.9	9.2 ± 0.7^*^	8.9 ± 0.8^*†#^	< 0.001
Phosphorous (mg/dL)	3.6 ± 0.6	4.0 ± 1.1^*^	4.2 ± 1.0^*^	4.5 ± 1.0^*†^	< 0.001
Calcium-phosphorous product (mg^2^/dL^2^)	34.2 ± 5.6	37.5 ± 9.4^*^	38.4 ± 8.1^*^	39.5 ± 8.9^*^	< 0.001
Uric acid (mg/dL)	8.4 ± 2.4	8.0 ± 2.0	8.2 ± 1.9	8.3 ± 2.0	0.497
PTH (pg/mL)	40.6 (29.6-60.8)	46.1 (26.6-110.2)	85.8 (49.5-173.3)^*†^	118.5 (59.3-222.4)^*†^	< 0.001
Upcr (mg/g)	166.5 (93.5-284.2)	864 (666-1030.6)^*^	1975 (1560-2322)^*†^	4950 (3435-8211)^*†#^	< 0.001
Medications					
ACEI and/or ARB use	67.2	57.3	53.8	56.1	0.164
Calcium-based phosphate binders	0	5.2	8.3	14.8^*^	0.003
Outcome					
eGFR slope (ml/min/1.73 m^2^/yr)	-1.31 (-2.16, -0.11)	-1.64 (-3.13, -0.72)	-3.18 (-5.03, -1.52)^*†^	-4.78 (-7.55, -2.42)^*†#^	< 0.001
eGFR slope < -3 ml/min/1.73 m^2^/yr (%)	14.7	25.8	52.1^*†^	69.9^*†#^	< 0.001
Progression to dialysis (%)	4.3	25.8^*^	47.1^*†^	60.2^*†^	< 0.001
Overall mortality (%)	8.6	18.5	18.5	25.2^*^	0.010
Cardiovascular mortality (%)	0.9	5.6	8.4	10.6^*^	0.015

Abbreviations. AoAC, aortic arch calcification; CTR, cardiothoracic ratio; eGFR, estimated glomerular filtration rate; CKD, chronic kidney disease; PTH, parathyroid hormone; Upcr, Urine protein-to-creatinine ratio; ACEI, angiotensin converting enzyme inhibitor; ARB, angiotensin II receptor blocker. The study patients were stratified into 4 groups according to quartiles of urine protein-to-creatinine ratio. ^*^p < 0.05 compared with quartile 1; ^†^*p* < 0.05 compared with quartile 2; ^#^*p* < 0.05 compared with quartile 3.

**Table 2:** Determinants of AoAC using multivariable stepwise linear regression analysis in study patients

Parameter	Multivariate (Stepwise)	
	Unstandardized coefficient β (95% CI)	*p*
Age (per 1 year)	0.017 (0.008, 0.026)	< 0.001
Calcium-phosphorous product (per 1 mg^2^/dL^2^)	0.020 (0.007, 0.033)	0.003
PTH (per 1 pg/mL)	-0.001 (-0.002,0)	0.040
Upcr (log per 1 mg/g)	0.315 (0.119, 0.511)	0.002

Values expressed as unstandardized coefficient β and 95% confidence interval (CI). Abbreviations are the same as in Table 1. Adjusted for age, gender, smoking, diabetes mellitus, hypertension, coronary artery disease, cerebrovascular disease, systolic and diastolic blood pressures, body mass index, fasting glucose, log-transformed triglyceride, total cholesterol, hemoglobin, baseline eGFR, total calcium, phosphorous, calcium-phosphorous product, uric acid, PTH, log-transformed U_PCR_, ACEI/ARB antihypertensive drug and calcium-based phosphate binders use.

**Table 3:** Determinants of CTR using multivariate stepwise linear analysis in study patients

Parameter	Multivariate (Stepwise)	
	Unstandardized coefficient β (95% CI)	*p*
Age (per 1 year)	0.087 (0.038, 0.135)	0.001
Male (*vs*. female)	2.953 (1.692, 4.213)	< 0.001
Coronary artery disease	1.944 (0.259, 3.630)	0.024
Cerebrovascular disease	3.728 (1.665, 5.790)	< 0.001
Body mass index (per 1 kg/m^2^)	0.210 (0.055, 0.365)	0.008
Hemoglobin (per 1 g/dL)	-0.364 (-0.677, -0.050)	0.023
Uric acid (per 1 mg/dL)	0.464 (0.185, 0.744)	0.001
Upcr (log per 1 mg/g)	1.186 (0.130, 2.242)	0.028
Calcium-based phosphate binders use	2.475 (0.175, 4.775)	0.035

Values expressed as unstandardized coefficient β and 95% confidence interval (CI). Abbreviations are the same as in Table 1. Adjusted for age, gender, smoking, diabetes mellitus, hypertension, coronary artery disease, cerebrovascular disease, systolic and diastolic blood pressures, body mass index, fasting glucose, log-transformed triglyceride, total cholesterol, hemoglobin, baseline eGFR, total calcium, phosphorous, calcium-phosphorous product, uric acid, PTH, log-transformed U_PCR_, ACEI/ARB antihypertensive drug and calcium-based phosphate binders use.

**Table 4:** Determinants of eGFR slope using multivariable stepwise linear regression analysis in study patients

Parameter	Multivariate (Stepwise)	
	Unstandardized coefficient β (95% CI)	*p*
Diastolic blood pressure (per 1 mmHg)	0.030 (0.005, 0.055)	0.019
Uric acid (per 1 mg/dL)	-0.185 (-0.651, -0.019)	0.029
Upcr (log per 1 mg/g)	-2.398 (-2.965, -1.831)	< 0.001

Values expressed as unstandardized coefficient β and 95% confidence interval (CI). Abbreviations are the same as in Table 1. Adjusted for age, gender, smoking, diabetes mellitus, hypertension, coronary artery disease, cerebrovascular disease, systolic and diastolic blood pressures, body mass index, fasting glucose, log-transformed triglyceride, total cholesterol, hemoglobin, baseline eGFR, total calcium, phosphorous, calcium-phosphorous product, uric acid, PTH, log-transformed U_PCR_, ACEI/ARB antihypertensive drug and calcium-based phosphate binders use.

**Table 5:** Relation of U_PCR_ quartiles and log-transformed U_PCR_ to progression to dialysis, overall and cardiovascular mortality using multivariate forward Cox proportional hazards model in study patients

Parameters	Commencement of dialysis		Overall mortality		Cardiovascular mortality	
	Hazard ratio (95% CI)	*p*	Hazard ratio (95% CI)	*p*	Hazard ratio (95% CI)	*p*
Model 1						
Quartile 1 of U_PCR_	Reference		Reference		Reference	
Quartile 2 of U_PCR_	3.476 (0.769-15.708)	0.105	1.844 (0.626-5.428)	0.267	3.853 (0.428-34.713)	0.229
Quartile 3 of U_PCR_	6.731 (1.531-29.600)	0.012	3.452 (1.179-10.109)	0.024	11.741 (1.422-96.935)	0.022
Quartile 4 of U_PCR_	6.639 (1.466-30.075)	0.014	4.845 (1.805-12.999)	0.002	12.974 (1.642-102.481)	0.015
Model 2						
U_PCR_ (log per 1 mg/g)	2.538 (1.375-4.685)	0.003	2.292 (1.329-3.953)	0.003	3.195 (1.393-7.325)	0.006

Values expressed as hazard ratio and 95% confidence interval (CI). Abbreviations are the same as in Table 1. Multivariate model 1: adjusted for age, gender, smoking, diabetes mellitus, hypertension, coronary artery disease, cerebrovascular disease, systolic and diastolic blood pressures, body mass index, fasting glucose, log-transformed triglyceride, total cholesterol, hemoglobin, baseline CKD stage, total calcium, phosphorous, calcium-phosphorous product, uric acid, PTH, log-transformed U_PCR_, ACEI/ARB antihypertensive drug and calcium-based phosphate binders use. Multivariate model 2: adjusted for age, gender, smoking, diabetes mellitus, hypertension, coronary artery disease, cerebrovascular disease, systolic and diastolic blood pressures, body mass index, fasting glucose, log-transformed triglyceride, total cholesterol, hemoglobin, baseline CKD stage, total calcium, phosphorous, calcium-phosphorous product, uric acid, PTH, log-transformed U_PCR_, ACEI/ARB antihypertensive drug and calcium-based phosphate binders use.
